# Group size effects and critical mass in public goods games

**DOI:** 10.1038/s41598-019-41988-3

**Published:** 2019-04-02

**Authors:** María Pereda, Valerio Capraro, Angel Sánchez

**Affiliations:** 1 Universidad Politécnica de Madrid, Departamento Ingeniería de Organización, Administración de empresas y Estadística, Madrid, Spain; 2 Unidad Mixta Interdisciplinar de Comportamiento y Complejidad Social (UMICC S), UC3M-UV-UZ Leganés, Madrid, Spain; 3 Economics Department, Middlesex University London, Business School, The Burroughs, London, NW4 4BT United Kingdom; 4 Grupo Interdisciplinar de Sistemas Complejos, Departamento de Matemáticas, Universidad Carlos III de Madrid, 28911 Leganés, Madrid, Spain; 5 Institute for Biocomputation and Physics of Complex Systems (BIFI), University of Zaragoza, 50018 Zaragoza, Spain; 6 Institute UC3M-BS for Financial Big Data (IBiDat), Universidad Carlos III de Madrid, 28903 Getafe, Madrid, Spain

**Keywords:** Social behaviour, Human behaviour

## Abstract

Understanding whether the size of the interacting group has an effect on cooperative behavior has been a major topic of debate since the seminal works on cooperation in the 1960s. Half a century later, scholars have yet to reach a consensus, with some arguing that cooperation is harder in larger groups, while others that cooperation is easier in larger groups, and yet others that cooperation attains its maximum in intermediate size groups. Here we add to this field of work by reporting a two-treatment empirical study where subjects play a Public Goods Game with a Critical Mass, such that the return for full cooperation increases linearly for early contributions and then stabilizes after a critical mass is reached (the two treatments differ only on the critical mass). We choose this game for two reasons: it has been argued that it approximates real-life social dilemmas; previous work suggests that, in this case, group size might have an inverted-U effect on cooperation, where the pick of cooperation is reached around the critical mass. Our main innovation with respect to previous experiments is that we implement a within-subject design, such that the same subject plays in groups of different size (from 5 to 40 subjects). Groups are formed at random at every round and there is no feedback. This allows us to explore if and how subjects change their choice as a function of the size of the group. We report three main results, which partially contrast what has been suggested by previous work: in our setting (i) the critical mass has no effect on cooperation; (ii) group size has a positive effect on cooperation; (iii) the most chosen option (played by about 50% of the subjects) is All Defection, followed by All Cooperation (about 10% of the subjects), whereas the rest have a slight trend to switch preferentially from defection to cooperation as the group size increases.

## Introduction

In the last decades, a variety of disciplines including anthropology, biology, economics, physics and psychology have addressed the issue of the inordinate scale of human cooperation^1–8^. Even in the simplest societies, individuals cooperate in groups of genealogically distant individuals^9,10^. In the perpetually connected society^11^ we live in today, instances requiring cooperation arise among groups of people of largely different sizes, and it is therefore of crucial importance to understand if and how group size affects cooperation. This question has been extensively debated in the literature since the seminal works on cooperation in the 1960s, leading to two different viewpoints. On the one hand, some scholars suggest that, in so far as the benefits of cooperation decrease with an increasing number of participants, cooperation should decrease with group size^12,13^. On the other hand, other researchers have argued that benefits do not necessarily decrease as the group size increases and, in that case, they reason that cooperation might even increase with the size of the group^14,15^. These two viewpoints have been mirrored by empirical, theoretical, and numerical works, with some finding a negative effect^16–22^, others finding a positive effect^22–31^, and yet others finding an ambiguous or non-significant effect^32–34^.

A commonly used, stylized setup to probe into the issue of cooperation is the Public Good Game^35^ (PGG). A PGG is a cartoon of the problem of provision of resources with three main characteristics: They are jointly provided, they are non-excludable (it is not possible to prevent those who have not paid for them from having access to them), and they are also non-rivalrous (resource consumption by one consumer does not prevent simultaneous consumption by others). Economic theory presents this as a game in which agents have to decide how much of their resources to contribute to the creation of a public good and how much to spend on private goods. The agents’ contribution is multiplied by some factor (linear PGG) and then distributed equally among everybody, irrespective of their own contribution. Even in such a simplified situation, the jury is still out on the issue of size dependence, with some researchers reporting an increase of contribution with the number of players^22–24^, and others finding no effects or negative effects^21,36^; a meta-analysis of 27 experiments^37^ concluded that there is mild positive effect of group size.

One way to make sense of these apparently contradictory results is by assuming that the effect of the group size on cooperation is not domain-general but depends on specific properties of the interaction. As suggested above, one potential discriminator is the way the benefit of full cooperation increases as a function of the group size. The intuition is simple: Since it is known that cooperation is positively related to the benefit of full cooperation^38,39^, if the benefit for full cooperation increases as the size of the group increases, then larger groups might get more tempted to cooperate; if, instead, the benefit for full cooperation decreases as the function of the group size, then larger groups might be less tempted to cooperate. Once again, from the theoretical viewpoint the issue is far from resolved. Thus, Peña considered evolutionary models, finding that the outcome of general nonlinear public goods games depends not only on the average group size but also on the variance of the group-size distribution in case groups are heterogeneous^40^; also, he showed that larger group sizes can have negative effects (by reducing the amount of cooperation in some cases) and positive effects (by enlarging the basin of attraction of more cooperative outcomes) on the evolution of cooperation^41^. Other works addressing multiplayer games from a more general but still evolutionary viewpoint point to the complexity of this problem^42–44^.

In order to gain insight into this problem, Capraro & Barcelo^45^ considered a class of *N*-person general public goods game parameterized by a function $\beta ({\rm{\Gamma }},N)$, representing the marginal return for cooperation when ${\rm{\Gamma }}$ people out of *N* cooperate. In the presence of ${\rm{\Gamma }}$ cooperators, the payoff of a cooperator is defined as $\beta ({\rm{\Gamma }},N)-c$ ($c > 0$ represents the cost of cooperation), while the payoff of a defector is defined as $\beta ({\rm{\Gamma }},N)$. In order to have a social dilemma, two properties are required:

- Full cooperation gives a larger payoff than full defection: $\beta (N,N)-c > \beta (0,0)$;
- Defecting is individually optimal, regardless of the number of cooperators: for all ${\rm{\Gamma }}\le N$, one has $\beta ({\rm{\Gamma }}-1,N) > \beta ({\rm{\Gamma }},N)-c$.

Barcelo & Capraro^22,45^ argue that, when $\beta ({\rm{\Gamma }},N)$ is constant or even decreasing in ${\rm{\Gamma }}$, then group size should have a negative effect on cooperation, whereas, when $\beta ({\rm{\Gamma }},N)$ is increasing in ${\rm{\Gamma }}$, then group size should have a positive effect on cooperation. They show that these two predictions are satisfied in two experiments and can be formalized by assuming that agents play according to the cooperative equilibrium model introduced by Capraro^46^. Putting these results together, it follows that, if we consider a piecewise linear-then-constant function $\beta ({\rm{\Gamma }},N)$, then group size should have a curvilinear, inverted-U effect on cooperation.

Note that public goods games with piecewise linear-then-constant return for cooperation are interesting because they approximate those real-life social dilemmas for which the public good has natural limits such that, from some point on, any new cooperator brings no additional benefit. For example, suppose that a hospital needs 50 liters of blood for the 50 victims of a terror attack. Of course, every litre of blood is initially important because it can save one life. However, after reaching the 50 litres, collecting more blood brings no additional benefit. Another example regards academic collaborations. Suppose that a team of researchers wants to start a multidisciplinary project that lies between three disciplines, for instance, psychology, anthropology and physics; and suppose that the team is looking for psychologists, anthropologists and physicists to take part to the project. Clearly, once each of these three disciplines is sufficiently covered, getting on board one more specialist would bring no additional benefit. The practical importance of this type of public goods games raises a crucial question: What is the group size effect on public goods games with a piecewise linear-then constant return for cooperation? Previous work using field experiments on real-life public goods games have repeatedly shown that medium-size groups tend to cooperate more^47–51^. In particular, connecting to one of our examples above, numerous studies have confirmed that, in academic research, the research quality of a research group is maximized by intermediate-size groups^52–54^. These findings are, thus, broadly consistent with the view that public goods game with a piecewise linear-then-constant return for cooperation should give rise to a inverted-U effect of group size on cooperation.

Following this theoretical prediction, Capraro & Barcelo^45^ carried out one experiment to probe into the existence of this curvilinear dependence of the group size. As predicted, they found evidence of an inverted-U dependence of the number of cooperators with the group size. However, while their prediction was that the rate of cooperation should start decreasing at $N=10$, it turned out that it reached its maximum in groups of size $N=15$. This immediately poses the question as to how the optimal group size for cooperation depends on the size at which *β*(*N*, *N*) becomes constant. This is an important question in light of possible applications of the curvilinear effect: knowing at which group size cooperation attains it maximum has obvious applications to team formation. In addition, a second question that was not answered by previous work regards the behavior of people at the individual level and its dependence on the group size. All the empirical studies on group size effect on cooperation that we are aware of uses a between-subjects design, such that different subjects interact in groups of different sizes. A major innovation of our work is that we implement a within-subject design, such that the same subject interacts in groups of different sizes, which will allow us to explore heterogeneity in people’s behavior as a function of the group size.

## Results

### Experimental setup

We conducted an online experiment with 200 subjects, that took place in eight sessions, one every two weeks between October 2017 and February 2018. The participants were Spanish volunteers from the IBSEN pool of subjects^55^. In each session, participants played one round of a *N*-person general public goods game with Critical Mass (i.e., $\beta ({\rm{\Gamma }},N)$ piecewise linear-then-constant). Sessions differed in the size of the interacting group, from $N=40$ in the first session to $N=5$ in the last one. Groups were randomly formed at the end of every session to compute the payoff. However, to avoid iterated game effects, participants received no feedback about the choices made by others or their own payoff until the last session was finished. To explore the influence on cooperation of the value where *β*(*N*, *N*) stabilizes (hereafter, *N*_*C*_), we carried out two different treatments, namely ${N}_{C}=10$ and ${N}_{C}=20$, each treatment with 100 participants initially assigned to it. Of these 200 volunteers we recruited, only 163 showed up the first day, having as a result a first day dropout of 18.5%. The experiment ended up with 107 volunteers that made all the decisions (average drop out rate of 5% per session). The experiment was implemented in IBSEN-oTree^55^. Participants played online through a web browser in a computer, tablet or mobile phone; and could made their decisions during eight hours each session day. Email reminders were sent every two weeks (i.e., before every session) to remind people to participate. Further details can be found in the Methods section. Only participants that completed the experiment were paid, as it was stated in the instructions. Participants were paid using PayPal. The average earning was 8 Euros. The 25% participants who scored highest in each treatment got a chance to enter a lottery of 50 Euros, and correspondingly two participants earned an additional price of 50 Euros.

### Data analysis: Cooperation as a function of the group size

As already indicated above, a total of 93 subjects (47.3% females) dropped out over the course of the experiment. Moreover, because of an internet connection problem, the condition $N=30$ was interrupted before data completion and, as a consequence, only 98 subjects participated in this condition. In view of these circumstances, the analysis is structured as follows: We first analyze subjects for whom we have all decisions and excluding the condition $N=30$. Subsequently, as robustness checks, we include also subjects who dropped out during the experiment and the condition $N=30$. As we will see below, the overall pattern of results is robust across these exclusions.

To carry out the analysis, we use the following variables: *C* is equal to 1 if a player cooperates, and 0 if a player defects; *N*_*C*_ is equal to 10 if a player participates in the condition where *β*(*N*, *N*) stabilizes at $N=10$, and equal to 20 if a player participates in the condition where *β*(*N*, *N*) stabilizes at *N* = 20; *N* is equal to the size of the group. Since *C* is a binary variable, the analysis will be conducted using a logistic model estimating the probability of cooperation *p*(*C*) as a function of the dependent variables ${x}_{1},\ldots ,{x}_{n}$ (which will be specified later):

1 $$p(C)=\frac{1}{1+{e}^{-({\alpha }_{0}+{\alpha }_{1}{x}_{1}+\ldots +{\alpha }_{n}{x}_{n})}}$$

**Figure 1:**
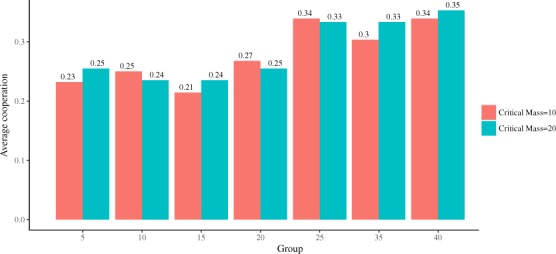
Average cooperation as a function of group size.

We begin by considering subjects for whom we have all the observations and excluding the condition $N=30$. In Fig. 1 we plot the percentage of people who cooperated. As a first step of the analysis, we observe that logistic regression predicting *C* as a function of *N*_*C*_ finds no statistically significant effect ($p=0.818$). Thus we can collapse data across the *N*_*C*_ treatments. Subsequently, logistic regression predicting *C* as a function of *N* finds a statistically significant positive effect, such that larger groups tend to cooperate more (coeff = 0.017, $z=2.45$, $p=0.014$). The aforementioned fact that *N*_*C*_ does not affect *C* is reflected in the fact that the positive effect of *N* on *C* is similar in the two treatments (${N}_{C}=10$: coeff = 0.016, $z=1.70$, $p=0.089$; ${N}_{C}=20$: coeff = 0.017, $z=1.86$, $p=0.078$).

We now turn to the robustness checks. As a first check, we repeat the analysis by including *N* = 30 in the data (19.44% cooperators in *N*_*C*_ = 10 and 21.62% cooperators in *N*_*C*_ = 20). As before, logistic regression predicting *C* as a function of *N*_*C*_ finds no statistically significant effect ($p=0.926$), and hence we collapse again data across the *N*_*C*_ treatments. In line with the previous results, we find a statistically significant positive effect of *N* on *C* (coeff = 0.014, $z=2.18$, $p=0.029$), which is similar in the two treatments (${N}_{C}=10$: coeff = 0.015, $z=1.54$, $p=0.123$; *N*_*C*_ = 20: coeff = 0.015, $z=1.54$, $p=0.123$). As a second robustness check, we include subjects who dropped out during the experiment, while excluding subjects who participated in *N* = 30. Once again, we find that *N*_*C*_ has no statistically significant effect on *C* ($p=0.424$) and, after collapsing data across the *N*_*C*_ treatments, we find a statistically significant positive effect of *N* on *C* (coeff = 0.016, $z=2.66$, $p=0.008$). Splitting the analysis by *N*_*C*_ reveals that the group size effect is significant only for ${N}_{C}=20$ (${N}_{C}=10$: coeff = 0.013, $z=1.53$, $p=0.125$; *N*_*C*_ = 20: coeff = 0.019, $z=2.21$, $p=0.027$). This suggests that the effect of group size might be stronger for ${N}_{C}=20$. However, the difference between the two effects is not statistically significant, as revealed by a logistic regression including the *N*_*C*_ × *N* interaction term ($p=0.897$). Therefore, also in this case we find that the group size effect does not depend on the critical mass. Finally, in our third and last robustness check, we analyze all data, that is, we include both subjects who dropped out during the experiment and those who participated in $N=30$. The results are qualilatively the same: *N*_*C*_ has no statistically significant effect on *C* ($p=0.398$), and when we collapse data across treatments *N* has a statistically significant positive effect on *C* (coeff = 0.015, $z=2.45$, $p=0.014$), which is similar in the two treatments (${N}_{C}=10$: coeff = 0.012, $z=1.38$, $p=0.169$; *N*_*C*_ = 20: coeff = 0.018, $z=2.07$, $p=0.039$), with the *N*_*C*_ × *N* interaction term turning out to be not statistically significant (p = 0.742). Therefore, our analysis combined with the three robustness checks we have considered, allows us to draw two main conclusions: First, the size of the group has a positive effect on cooperation, and second, the positive effect of group size on cooperation does not depend on the critical mass. We have also conducted the same analyses by adding a quadratic term for the group size effect, in order to test for the inverted-U effect. Results remain qualitatively the same, and the quadratic term is never significant.

One additional step we have taken to assess the reliability of the above findings is to study separately the average cooperation among subjects who dropped out during the experiments (44 in the treatment ${N}_{C}=10$ and 49 in the treatment ${N}_{C}=20$), by comparing it with the average cooperation among subjects who did not drop out. The idea behind this check is that, as the experiment proceeded, it could be the case that individuals more prone to cooperation were overrepresented among the dropouts, thus leading to a decrease in average cooperation as the experiment proceeded from the largest to the smallest size. To test for this possibility, we built a dummy variable named *Incomplete*, which takes value 1 if a subject dropped out during the experiment, and 0 if a subject completed the experiment. We then conducted a set of logistic regressions predicting *C* as a function of *Incomplete*, for each value of *N*. In doing so, we find all the p-values to be larger than 0.2. This suggests that the average cooperation among those who dropped out during the experiment is not statistically different than the average cooperation among those who completed the experiment, which in turn supports the conclusion that our results are unlikely to be driven by the relatively high rate of dropping out.

### Within-subject analysis

In the previous subsection, we have looked at our experiment from the viewpoint of the global results on cooperation and its dependence on the group size. Now we move on to studying heterogeneity in people’s behavior. We begin by observing that, since each participant has to take eight independent decisions about whether to cooperate or not, there are ${2}^{8}=256$ possible combinations of cooperation and defection. However, as summarized in Table 1, for each *N*_*C*_ treatment, only 25 of them, less than 10%, are actually being played.

**Table 1:** Frequency (and number of subjects) playing each strategy, for treatments *N*_*C*_ = 10 and *N*_*C*_ = 20.

Critical Mass = 10			Critical mass = 20		
Strategy	#Subjects	Frequency	Strategy	#Subjects	Frequency
DDDDDDDD	20	0.3571	DDDDDDDD	20	0.3922
DD_DDDDD	7	0.1250	DD_DDDDD	5	0.0980
CCCCCCCC	3	0.0536	CCCCCCCC	3	0.0588
DC_CCCCC	2	0.0357	DCDDDDDD	2	0.0392
CDDDDDDD	2	0.0357	DDDDDDCD	1	0.0196
CD_CCCCC	2	0.0357	DDCDDDDD	1	0.0196
CC_CCCCC	2	0.0357	DCDCDDDD	1	0.0196
DDDDDDDC	1	0.0179	DCDCCCDD	1	0.0196
DDDCDDDD	1	0.0179	DCCDCCDC	1	0.0196
DD_DCDDD	1	0.0179	DC_CCCCC	1	0.0196
DCDDCDDD	1	0.0179	CDDDDDDD	1	0.0196
DCDCCCCC	1	0.0179	CDDDCDDD	1	0.0196
DCCCDDCD	1	0.0179	CDCCDCCC	1	0.0196
DC_DDDDD	1	0.0179	CD_DCDDC	1	0.0196
DC_DCCCC	1	0.0179	CD_CDDDD	1	0.0196
CDDDDDCD	1	0.0179	CD_CCDDD	1	0.0196
CDDCDCDD	1	0.0179	CD_CCCCC	1	0.0196
CDCCCDCC	1	0.0179	CCDCDDDC	1	0.0196
CD_DDDDD	1	0.0179	CCDCCDCC	1	0.0196
CD_CDCDD	1	0.0179	CCCCDCCC	1	0.0196
CCDDDDDD	1	0.0179	CCCCCCDD	1	0.0196
CCCCDDDD	1	0.0179	CC_DDDCC	1	0.0196
CCCCCDDD	1	0.0179	CC_CDDCD	1	0.0196
CC_DDDCD	1	0.0179	CC_CDCDD	1	0.0196
CC_CCDDC	1	0.0179	CC_CCCCC	1	0.0196

D stands for Defection, C stands for Cooperation.

**Figure 2:**
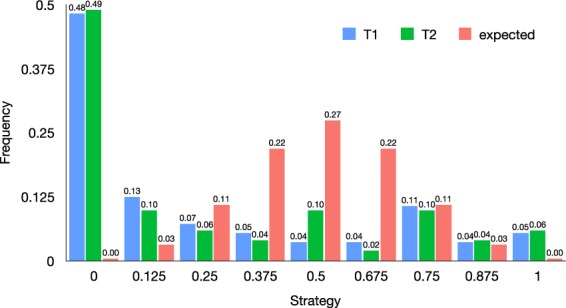
The *x* axis reports the frequency with which cooperation appears in a given sequence of choices (e.g., $x=0$ corresponds to All Defection, whereas $x=1$ corresponds to All Cooperation). The *y* axis reports the frequency of people playing strategies with a given proportion of cooperative strategies, for treatments T1 and T2, versus the expected frequency of each strategy is they were chosen at random.

Not surprisingly, in both treatments ${N}_{C}=10$ and ${N}_{C}=20$, the most played strategy is AllD (All Defection), making a total of about 48% of subjects in the treatment ${N}_{C}=10$ (counting also subjects who did not participate in condition $N=30$), and 49% of subjects in the treatment ${N}_{C}=20$. Perhaps more surprisingly, in both treatments, the second most played strategy, albeit at a large distance from the first, is AllC (All Cooperation), which makes about 9% of the observations in the treatment ${N}_{C}=10$ and 8% of the observations in the treatment ${N}_{C}=20$ (again counting also subjects who did not participated in the session with $N=30$). All other strategies have been played by less than 4% of the participants. For comparison, Fig. 2 shows the distribution of sequences we would expect if the decisions were taken at random. Each “strategy” groups the set of sequences with the same rate of cooperation; for example, cooperating once and defecting 7 times is “strategy 0.125”. The plot makes it evident that participants’ choices are largely intentional: People play AllD far more than expected, and the most expected strategies arising from random choices are barely played. Only highly cooperative strategies (0.75, 0.875) appear to a similar extent to what would be expected by chance, except for AllC, which appears to be more frequent.

**Figure 3:**
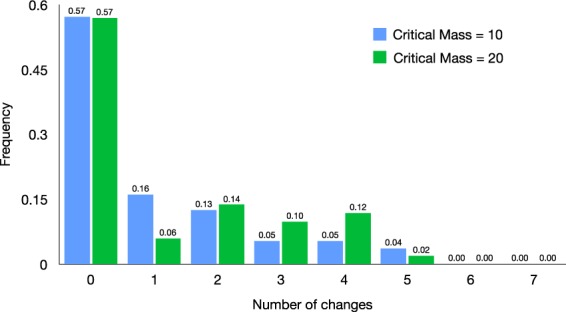
Frequency of people as a function of the number of changes in their sequence of decisions, for treatments *N*_*C*_ = 10 and *N*_*C*_ = 20.

Therefore, about half of the participants played the Nash equilibrium of the game, choosing to defect in all sessions, irrespective of the value of the critical mass *N*_*C*_. On the other hand, as a consequence of the fact that the majority of the subjects do not change strategy, the average number of times a player changes strategy over the course of the eight sessions is relatively low (1.08 out of maximum of 7). In fact, only 43% of the participants changed their decision at least once: As shown in Fig. 3, there are subjects who change strategy even five times, but no one changes strategy more than that.

We now explore in more detail the sequence of actions taken by subjects who changed their decision at least once in the eight sessions. Across the two *N*_*C*_ treatments and among the 12 subjects who change strategy only once, 8 of them (67%), move from C to D, while only 33% move from D to C, i.e., half of those who changed in the opposite direction. Fourteen subjects change their choice twice, accounting for 28 option changes. 43% of them go from C to D and back, and 57% do the opposite. As for the rest of the changes, a total of 75, 34 of them (45%) are switches from D to C, and the remaining 41 go from C to D. Thus, the only net effect of the changes seems to be in the subjects who changed only one, a behavior that could be a possible reason for the observed increment in cooperation with size. However, because of the small sample size, this should be read as a hint to be confirmed in future work, rather than a well-established conclusion.

Finally, we also checked for potential gender effects, especially because previous research suggests that gender may be related to prosocial preferences in a non-trivial way. In particular, Croson & Gneezy^56^ argue that women are more altruistic than men in the Dictator game, but that there are no stable gender differences in cooperation games such as the Prisoner’s dilemma and the Public Goods game. On the contrary, Molina *et al*.^57^ find some evidence supporting larger cooperativeness among female high school students. Two recent meta-analyses^58,59^ found that women are more altruistic than men, especially when acting under intuition^59^, and that gender differences in cooperation, if existing, are likely to be very small^60^. In line with this general picture, also in our cooperation problem, we found no gender differences in cooperation. Specifically, we conduct a set of logistic regressions predicting *C* as a function of *Female*, for each value of *N* and also collapsing all group sizes together, finding all p-values to be larger than 0.1.

## Discussion

Here we have contributed to the ongoing debate regarding the effect of group size on cooperative behavior. We have experimentally investigated the possibility of observing nonlinear dependencies of cooperation with group size in public goods game with a critical mass. Our specific research questions related to the possibility, predicted theoretically, of observing a maximum in cooperation as a function of the group size around the critical mass at which additional cooperators do not lead to increase public good production.

In contrast to this prediction, we observe a monotonous increase of the rate of cooperation as a function of the group size. Furthermore, our results suggest that the critical mass has no influence on the rate of cooperation. In addition, we have also looked at heterogeneity in individual behavior, finding approximately half of the subjects never contributing, another 10% that were unconditional cooperators, whereas the rest show a tendency to change preferentially from defection to cooperation than the converse, as the size of the group increases.

There are several reasons why we may have not observed an inverted-U effect of group size on cooperation in our experiment. One might have been selection bias: it is possible that subjects who dropped out in the middle of the experiment were high cooperators with respect to those who completed the experiment, and this might have killed the pick of cooperation at intermediate-size groups. However, as we have seen in the Results section, our analysis shows that subjects who dropped out cooperated at the same rate as those who completed the experiment, thus ruling out this possible explanation. Another possible reason for the discrepancy comes from a closer, data-driven, inspection of the results reported by Capraro and Barcelo^45^. Here, the maximum rate of cooperation was observed for $N=15$. However Fig. 1 in Capraro & Barcelo^45^ highlights that the case $N=15$ gave rise to an unusually high rate of cooperation, suggesting that it might have been an outlier. Treating $N=15$ in Capraro & Barcelo^45^ as an outlier results in a broad maximum around $N=25$, that could only be noticed by going to groups as large as about 100 people. If the maximum rate of cooperation in public goods game with a critical mass is attained this far from the critical mass for the case ${N}_{C}=10$, the situation could be further complicated in the case ${N}_{C}=20$, in which the rate of cooperation could reach its maximum for group sizes not considered in our experiment — contributing to mask the effect. It is also possible that the payoff structure of the game affects the results, as has been observed, e.g., by Nosenzo *et al*.^21^, who observed no group size effect for low marginal per capita returns and negative effect of group size for high marginal per capita returns. Another potential explanation is that within-subject experiments give rise to fundamentally different dynamics than between-subjects ones, perhaps for the following reason. In order to have an inverted-U effect of the group size on cooperation in a within subject design, one would need a significant proportion of subjects that change strategy twice: from defection, to cooperation, and then back to defection. This somehow conflicts with the literature suggesting that subjects have preferences for being consistent^61^, especially when it comes to costly prosocial behavior^62^. In line with this view, our analysis of the heterogeneity in people’s behavior shows that about 60% of the subjects never change strategy, and about 70% of the subjects change strategy only once. This might have partly contributed to the lack of an inverted-U effect of group size on cooperation. The discrepancy could perhaps be understood in terms of evolutionary game theory, because from that view point the direction and size of the effect could depend on external control parameters such as the mutation rate or the intensity of the selection^5^. Finally, reminding that the existence of an inverted-U effect was deduced by assuming that subjects play according to the cooperative equilibrium model^46^, it is possible that, in our setting, this model does not represent a good approximation of subjects’ behavior. Although this might be true in general, it would not explain why we found a positive effect of group size on cooperation, while Capraro & Barcelo^45^ found an inverted-U effect. Further experimental work, involving much larger number of subjects and groups, is needed to discriminate among these options and have a more complete understanding of if, when, and how group size impacts cooperative behavior.

## Methods

All participants in the experiments reported in the manuscript signed an informed consent to participate when enrolling in the IBSEN volunteer pool^55^. In agreement with the Spanish Law for Personal Data Protection, their anonymity was always preserved. This procedure was approved by the Ethics Committee of Universidad Carlos III de Madrid, the institution responsible for funding the experiment, and the experiment was subsequently carried out in accordance with the approved guidelines.

**Figure 4:**
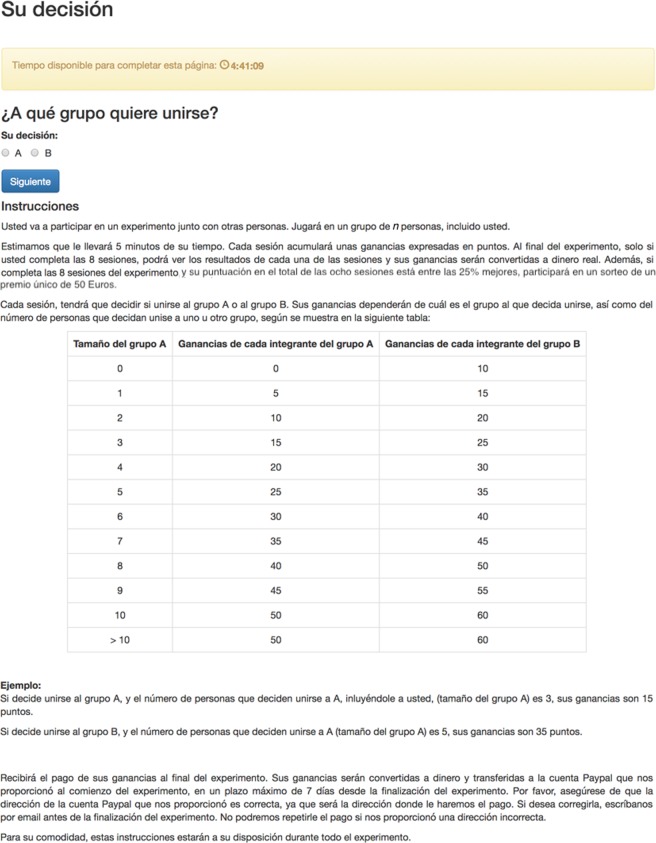
Screenshot of decision screen for the condition *N*_*C*_ = 10.

The experimental instructions were prepared following as closely as possible those used in^45^, albeit translated into Spanish. A screenshot of the decision page is shown in Fig. 4. Here we include a translated version of the instructions:

“*You are going to participate in an experiment with other people*. *You will play in groups of N people*.

*It will take you about 5* *minutes*. *Each wave*, *your earnings will be shown in points*, *and only if you complete the 8 waves of the experiment*, *these earnings will be converted to money*. *Additionally*, *if you complete the eight sessions of the experiment and your total score* (*of the eight sessions*) *is among the 25*% *best*, *you will participate in a lottery for a single prize of 50 Euros*.

*Each session*, *you will have to decide to join either Group A or Group B*. *Your earnings will depend on the group you decide to join and on the size of the two groups*, *A and B*, *as follows*:

*Example*:

*If you join A*, *and the number of people that also decided to join A* (*size A*) *is 3 people*, *you will earn 15 points*.

*If you join B*, *and the number of people that decided to join A* (*size A*) *is 5 people*, *you will earn 35 points*.

*You will be paid at the end of the experiment*. *Your earnings will be converted to money and transferred to your PayPal account*, *in a maximum period of 7 days counting from the end of the experiment*. *Please make sure the address of the Paypal account you provide us is correct*, *since it will be the address used for the payment*. *If you want to correct it*, *please write to us by email before the end of the experiment*. *We will not be able to repeat the payment if the account address is incorrect*. *For your convenience*, *these instructions will be available throughout the experiment*.”

At the beginning of each session, the instructions were shown, and afterwards participants had to complete four control questions to facilitate the understanding of the experiment. They had five opportunities to give the right answer, and afterwards, the correct answer was shown. The questions were:

- Q1: Imagine you decide to join group A, and the number of people that also decided to join A (size A) is 4 people. How many points will you earn?
- Q2: Imagine you decide to join group B, and the number of people that also decided to join A (size A) is 8 people. How many points will you earn?
- Q3: Imagine you decide to join group A, and the number of people that also decided to join A (size A) is 15 people. How many points will you earn?
- Q4: Imagine you decide to join group B, and the number of people that decided to join A (size of A) is 20 people. How many points will you earn?

During the experiment, the only information available to the participants was the group size they were playing every time and the beta function (as a function of A and B decisions) presented by means of Table 2. Participants played online through a web browser in a computer, tablet or mobile phone; and could make their decisions during eight hours each session day. After each of the sessions, which where all one-shot game sessions, groups were randomly formed only with participants that made their decision in order to compute the payoffs. Participants membership to these randomly formed groups (of size *N*) did not remain constant across sessions, but new random groupings were created after each session. For participants in incomplete groups, we assigned a payoff by randomly sampling among participants in the corresponding session that made the same decision (cooperate/not cooperate). The payoffs of all rounds were only shown at the end of the experiment.

**Table 2:** Payoff beta functions showed in the experiment: *N*_*C*_ = 10 (left) and *N*_*C*_ = 20 (right).

Size of Group A	Payoff of A	Payoff of B
0	0	10
1	5	15
2	10	20
3	15	25
4	20	30
5	25	35
6	30	40
7	35	45
8	40	50
9	45	55
10	50	60
>10	50	60
0	0	10
1	5	15
2	10	20
3	15	25
4	20	30
5	25	35
6	30	40
7	35	45
8	40	50
9	45	55
10	50	60
11	55	65
12	60	70
13	65	75
14	70	80
15	75	85
16	80	90
17	85	95
18	90	100
19	95	105
20	100	110
>20	100	110

### Accession codes

Data is available in an structured way at Zenodo public repository with 10.5281/zenodo.2565197.
