# Author Correction: Group size effects and critical mass in public goods games

**DOI:** 10.1038/s41598-019-49306-7

**Published:** 2019-09-03

**Authors:** María Pereda, Valerio Capraro, Angel Sánchez

**Affiliations:** 1 Universidad Politécnica de Madrid, Departamento Ingeniería de Organización, Administración de empresas y Estadística, Madrid, Spain; 2 Unidad Mixta Interdisciplinar de Comportamiento y Complejidad Social (UMICC S), UC3M-UV-UZ, Leganés, Madrid, Spain; 3 Economics Department, Middlesex University London, Business School, The Burroughs, London, NW4 4BT United Kingdom; 4 Grupo Interdisciplinar de Sistemas Complejos, Departamento de Matemáticas, Universidad Carlos III de Madrid, 28911, Leganés, Madrid, Spain; 5 Institute for Biocomputation and Physics of Complex Systems (BIFI), University of Zaragoza, 50018 Zaragoza, Spain; 6 Institute UC3M-BS for Financial Big Data (IBiDat), Universidad Carlos III de Madrid, 28903, Getafe, Madrid, Spain

Correction to: *Scientific Reports* 10.1038/s41598-019-41988-3, published online 02 April 2019

This Article contains an error in Reference 41.

Peña, J. Group size effects in social evolution. *J. Theor. Biol*. **457**, 211–220 (2018).

should read:

Peña, J. & Nöldeke, G. Group size effects in social evolution. *J. Theor. Biol*. **457**, 211–220 (2018).

As a result, in the Introduction section,

“Thus, Peña considered evolutionary models, finding that the outcome of general nonlinear public goods games depends not only on the average group size but also on the variance of the group-size distribution in case groups are heterogeneous^40^; also, he showed that larger group sizes can have negative effects (by reducing the amount of cooperation in some cases) and positive effects (by enlarging the basin of attraction of more cooperative outcomes) on the evolution of cooperation^41^.

should read:

“Thus, Peña considered evolutionary models, finding that the outcome of general nonlinear public goods games depends not only on the average group size but also on the variance of the group-size distribution in case groups are heterogeneous^40^; also, he and Nöldeke showed that larger group sizes can have negative effects (by reducing the amount of cooperation in some cases) and positive effects (by enlarging the basin of attraction of more cooperative outcomes) on the evolution of cooperation^41^.”

